# Non-B, Non-C Hepatocellular Carcinoma in an HBV- and HCV-Endemic Area: A Community-Based Prospective Longitudinal Study

**DOI:** 10.3390/v14050984

**Published:** 2022-05-07

**Authors:** Te-Sheng Chang, Nien-Tzu Hsu, Shu-Chuan Chen, I-Lin Hsu, Mei-Hsuan Lee, Sheng-Nan Lu

**Affiliations:** 1 Department of Gastroenterology and Hepatology, Division of Internal Medicine, Chang Gung Memorial Hospital, Chiayi 613016, Taiwan; cgmh3621@cgmh.org.tw; 2 College of Medicine, Chang Gung University, Taoyuan 333323, Taiwan; 3 Biostatistics Center of Kaohsiung Chang Gung Memorial Hospital, Kaohsiung 833253, Taiwan; e19911221@gmail.com; 4 Public Health Bureau, Tainan City Government, Tainan 701017, Taiwan; hp01@tncghb.gov.tw (S.-C.C.); hsu@tncghb.gov.tw (I.-L.H.); 5 School of Medicine, National Yang Ming Chiao Tung University, Taipei 112304, Taiwan; 6 Department of Gastroenterology and Hepatology, Division of Internal Medicine, Kaohsiung Chang Gung Memorial Hospital, Kaohsiung 833401, Taiwan

**Keywords:** non-B, non-C, hepatocellular carcinoma, risk factor, prospective study

## Abstract

A large community cohort of adults who participated in a health screening program from 2003 to 2013 were prospectively analyzed for the risk factors of non-B, non-C (NBNC) hepatocellular carcinoma (HCC). The serostatus of hepatitis B and C of 52,642 participants was linked to the mortality and cancer registration data of the Health and Welfare Data Science Center, Ministry of Health and Welfare, Taiwan. During a median follow-up of 6 years, 35 of the 43,545 participants who were negative for both HBsAg and anti-HCV antibody developed HCC. Multivariate Cox regression analysis revealed that old age (hazard ratio, 95% CI: 1.058, 1.019–1.098, *p* = 0.003); male sex (2.446, 1.200–4.985, *p* = 0.014); high aspartate aminotransferase levels (6.816, 2.945–15.779, *p* < 0.001); fibrosis index based on four factor score (1.262, 1.154–1.381, *p* < 0.001); blood sugar (1.009, 1.002–1.015, *p* = 0.006); and alpha-fetoprotein ≥15 ng/mL (143.938, 43.094–480.760, *p* < 0.001) were independent risk factors for HCC. By contrast, triglyceride >150 mg/dL was associated with a decreased risk of HCC (0.216, 0.074–0.625, *p* = 0.005). This prospective community-based study provided insights into the potential HCC risk factors which may shed some light in HCC prevention and screening.

## 1. Introduction

Hepatocellular carcinoma (HCC), which generally arises in the setting of underlying chronic liver disease, is the sixth most common cancer and the third leading cause of cancer-related death worldwide after lung and colorectal cancer [1]. The incidence and distribution of HCC vary greatly according to geographic location and ethnic group [2,3]. Regional variations in exposure to various risk factors are attributable to these extreme differences in the etiologies of HCC [4]. Chronic infection with hepatitis B virus (HBV) and/or hepatitis C virus (HCV) is the most common cause of chronic liver disease and the subsequent development of liver cirrhosis and HCC [5]. However, there is a substantial number of HCC patients negative for both markers of HBV and HCV infection, referred to as non-B, non-C HCC (NBNC HCC). With the emerging availability of measures for treating and controlling viral hepatitis, the role of HBV and HCV in the development of HCC is decreasing [6,7]. In contrast, recent studies have revealed that the proportion and number of patients with NBNC HCC are on the rise [8,9].

In addition to viral hepatitis, multiple metabolic, environmental, and genetic factors have been linked to NBNC HCC [8,10,11,12,13,14]. The prediction of individual HCC risk is critical for the implementation of effective and feasible HCC screening and surveillance programs. Identifying the etiologies and risk factors for NBNC HCC is important for the future development of HCC prediction models that can be used in clinical practice [15]. Individualized risk-based approaches for preventive strategies, encompassing HCC screening and chemopreventive intervention, will not be successful or feasible without detailed knowledge of HCC etiologies or risk factors. Compared to virus-related HCC, little is known about the characteristics of NBNC HCC, and the screening and surveillance programs for HCC of this category are not well established [16,17]. Without a consensus on the mode of screening and surveillance, NBNC HCC is usually found incidentally and the tumor stage is generally late at the time of diagnosis, which may lead to a poorer outcome for NBNC HCC in comparison to virus-related HCC [18,19,20,21]. To reduce this forthcoming global disease burden of NBNC HCC, it is quite important to clarify its potential etiologies and risk factors.

A handful of studies have been carried out to explore the etiologies and clinical features of NBNC HCC [8,9,10,11,12,13,14,18,19,20,22,23,24]. However, almost all prior reports analyzing the characteristics of NBNC HCC were either hospital-based or retrospective studies. This study examined the association between HCC occurrence and selected nonviral factors frequently checked on routine measurements or screening programs in a community-based prospective manner.

## 2. Materials and Methods

### 2.1. Case Enrollment and Data Organization

In Taiwan, all adults are eligible for, and are encouraged to participate in, a government-funded health promotion program every 3 years if they are ≥40 years old and annually if they are ≥65 years old. The investigation items selected for this program are evidence-based and scrutinized by expert panels focusing on 6 major health measures, including blood pressure, blood sugar, lipid profile, body weight and height, as well as renal and liver function. In addition to general demographic and health-related behavior data, laboratory tests including albumin, globulin, aspartate aminotransferase (AST), alanine aminotransferase (ALT), blood urine nitrogen (BUN), creatinine, uric acid, fasting blood sugar, total cholesterol, triglyceride, high-density lipoprotein cholesterol (HDL-C), low-density lipoprotein cholesterol (LDL-C), and a hemogram are obtained at each time of measurement. Hepatitis B surface antigen (HBsAg), anti-HCV antibody (anti-HCV), and alpha-fetoprotein (AFP) were tested at the first time of participation.

**Figure 1:**
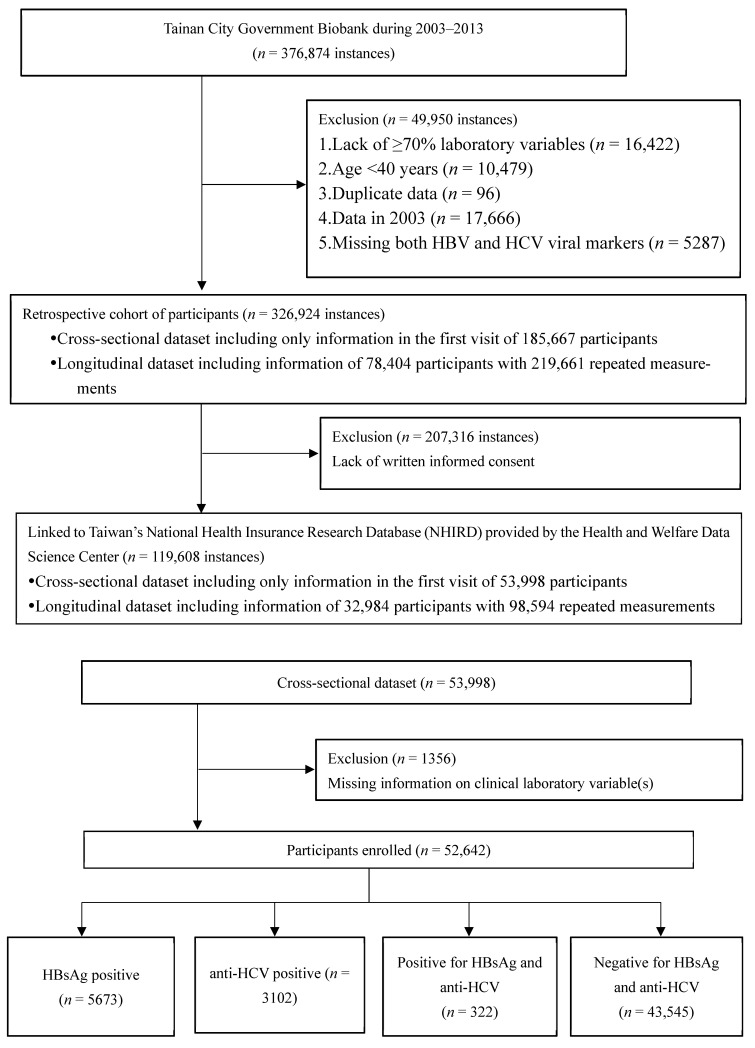
Flow diagram showing the disposition of participants in the study.

From 2003 to 2013, there were 376,874 instances of participation in this program in Tainan, a municipality in southern Taiwan. These health datasets were digitized and serum specimens were stored in Tainan City Government Biobank. Considering data integrity and accuracy, 17,666 datasets obtained in the first year were omitted. Duplicate data (*n* = 96), data from participants aged <40 years (*n* = 10,479), data lacking ≥70% of laboratory variables (*n* = 16,422), and data missing both HBV and HCV viral markers (*n* = 5287) were also excluded. Among the remaining 326,924 participations, 185,667 participants received a checkup only once, while 78,404 participants received two or more checkups and comprised a longitudinal cohort with a total of 219,661 measurements. All variables obtained from all enrolled participants for the first time during this period were used as the indicators. After excluding those lacking written informed consent (*n* = 207,316 instances), 119,608 datasets were included and accessed to the Health and Welfare Data Science Center (HWDC), Ministry of Health and Welfare. Those without complete information on clinical laboratory variable(s) (*n* = 1356) were further excluded. Finally, 52,642 participants were enrolled in this study, as demonstrated in Figure 1. The participants were stratified according to the serostatus of hepatitis B and C, namely, HBsAg positive (HBV group), anti-HCV positive (HCV group), dual HbsAg and anti-HCV positive (BC group), and negative for both HBsAg and anti-HCV (NBNC group). The data were linked to the overall mortality and cancer registration data of the HWDC, which was implemented in 1978. All data were decoded to prevent the identification of individual participants. This study was approved by the Institutional Review Board of Chang Gung Medical Foundation on 15 April 2019 (IRB No.: 102-5754B) and was conducted in accordance with the principles of Declaration of Helsinki and the International Conference on Harmonization for Good Clinical Practice.

### 2.2. Statistical Analysis

All continuous variables are expressed as the mean ± standard deviation (SD). Differences between 2 groups were analyzed using chi-square test for dichotomous variables and Student’s *t*-test for continuous variables. Multivariate Cox regression models were employed to explore the relative contributions of various factors to the occurrence of HCC. Differences among 3 different groups were compared with one-way analysis of variance (ANOVA) with Bonferroni’s pairwise comparison. The incidence of HCC and the cumulative survival of participants were determined using Kaplan–Meier analysis. All statistical analyses were performed using SAS software (Version 9.4, SAS Institute Inc., Cary, NC, USA). A two-tailed *p*-value of <0.05 was considered significant.

## 3. Results

### 3.1. Baseline Case Characteristics

Among the 52,642 participants enrolled in this study, 5673 (10.8%) were positive for HBsAg, 3102 (5.9%) were positive for anti-HCV, 322 (0.6%) were positive for both HBsAg and anti-HCV, and 43,545 (82.7%) were negative for both HBsAg and anti-HCV. Over a median follow-up of 6 years, 228 participants developed HCCs, with 72 (1.3%), 109 (3.5%), 12 (3.7%), and 35 (0.1%) in the HBV, HCV, BC, and NBNC groups, respectively. Table 1 demonstrates the baseline characteristics of the 43,545 participants in the NBNC group. When compared to the 43,510 cases without HCC, the 35 cases with HCC were older (65.0 ± 9.5 vs. 56.9 ± 10.0 years, *p* < 0.001); male predominant (*p* = 0.005); had higher body mass index (BMI) (25.8 ± 3.5 vs. 24.4 ± 3.5 kg/m^2^, *p* = 0.019), blood sugar (112.3 ± 35.7 vs. 96.8 ± 29.8 mg/dL, *p* = 0.002), uric acid (6.3 ± 1.6 vs. 5.7 ± 1.6 mg/dL, *p* = 0.028), AST (39.0 ± 30.7 vs. 24.9 ± 11.7 U/L, *p* = 0.010), ALT (34.2 ± 20.4 vs. 25.2 ± 18.4 U/L, *p* = 0.004), AST to platelet ratio index (APRI) score (0.63 ± 0.85 vs. 0.29 ± 0.19, *p* = 0.022), and fibrosis index based on four factors (FIB4) score (2.57 ± 2.23 vs. 1.38 ± 0.70, *p* = 0.003); and had lower platelet count (202.6 ± 70.6 vs. 233.6 ± 60.7 × 10^3^ cells/µL, *p* = 0.003) and triglycerides (103.8 ± 50.1 vs. 132.4 ± 99.0 mg/dL, *p* = 0.002). Baseline characteristics of the participants in the HBV, HCV, and BC groups are demonstrated in Supplementary Tables S1–S3, respectively. Briefly, HBV HCC individuals were older (59.8 ± 9.9 vs. 55.5 ± 9.0 years, *p* < 0.001); male predominant (*p* < 0.001); had higher creatinine (1.1 ± 0.2 vs. 1.0 ± 0.2 mg/dL, *p* = 0.005), AST (50.4 ± 53.2 vs. 28.6 ± 19.9 U/L, *p* < 0.001), ALT (47.2 ± 46.9 vs. 31.0 ± 35.2 U/L, *p* = 0.005), APRI score (0.97 ± 1.18 vs. 0.37 ± 0.34, *p* < 0.001), and FIB4 score (3.18 ± 2.45 vs. 1.53 ± 0.87, *p* < 0.001); and lower platelet count (160.2 ± 56.5 vs. 214.2 ± 57.3 × 10^3^ cells/µL, *p* < 0.001) and total cholesterol (186.5 ± 37.3 vs. 202.1 ± 35.9 mg/dL, *p* < 0.001). HCV HCC individuals were older (67.1 ± 5.9 vs. 61.8 ± 9.2 years, *p* < 0.001); male predominant (*p* < 0.001); had more hypertension (*p* = 0.024); had higher creatinine (1.2 ± 0.3 vs. 1.1 ± 0.3 mg/dL, *p* = 0.002), AST (78.8 ± 52.1 vs. 41.1 ± 48.4 U/L, *p* < 0.001), ALT (87.9 ± 61.0 vs. 46.2 ± 62.9 U/L, *p* = 0.005), APRI score (1.67 ± 1.42 vs. 0.63 ± 0.90, *p* < 0.001), and FIB4 score (4.67 ± 2.99 vs. 2.24 ± 1.54, *p* < 0.001); and lower platelet count (144.8 ± 56.4 vs. 195.6 ± 58.9 × 10^3^ cells/µL, *p* < 0.001) and total cholesterol (175.3 ± 36.7 vs. 193.2 ± 37.9 mg/dL, *p* < 0.001). The BC HCC group was male predominant (*p* = 0.005) compared to the non-HCC counterparts.

### 3.2. Characteristics and Factors Associated with HCC Development

As shown in Table 2, factors associated with HCC development in the 43,545 NBNC participants included old age (hazard ratio (HR): 1.081, 95% confidence interval (CI): 1.043–1.120, *p* < 0.001), male gender (2.787, 1.385–5.608, *p* = 0.004), BMI 25–30 kg/m^2^ (2.323, 1.155–4.671, *p* = 0.018), AST > 45 U/L (9.330, 4.236–20.550, *p* < 0.001), ALT > 45 U/L (2.570, 1.066–6.191, *p* = 0.035), high uric acid (1.250, 1.035–1.510, *p* = 0.021), AFP ≥ 15 ng/mL (99.519, 30.377–326.041, *p* < 0.001), platelet count < 150 × 10^3^ cells/µL (5.383, 2.440–11.876, *p* < 0.001), high APRI (1.524, 1.309–1.775, *p* < 0.001), high FIB4 score (1.268, 1.196–1.345, *p* < 0.001), and high blood sugar (1.009, 1.003–1.014, *p* = 0.001). Triglyceride > 150 mg/dL had an inverse relationship with HCC development (0.351, 0.124–0.994, *p* = 0.049). Multivariate Cox regression analysis revealed that old age (1.058, 1.019–1.098, *p* = 0.003); male gender (2.446, 1.200–4.985, *p* = 0.014); BMI 25–30 kg/m^2^ (2.480, 1.203–5.112, *p* = 0.014); high AST (6.816, 2.945–15.779, *p* < 0.001), FIB4 score (1.262, 1.154–1.381, *p* < 0.001), and blood sugar (1.009, 1.002–1.015, *p* = 0.006); and AFP ≥ 15 ng/mL (143.938, 43.094–480.760, *p* < 0.001) were independent risk factors for HCC development. On the contrary, triglyceride > 150 mg/dL was independently associated with a decreased risk (0.216, 0.074–0.625, *p* = 0.005). Characteristics and factors associated with HCC development of the participants in the HBV, HCV, and BC groups are demonstrated in Supplementary Tables S4–S6, respectively. For HBV HCC development, male gender (2.848, 1.628–4.982, *p* < 0.001), high high-density lipoprotein cholesterol (1.024, 1.009–1.040, *p* = 0.002), high FIB4 score (1.283, 1.197–1.375, *p* < 0.001), high platelet count < 150 ×10^3^ cells/µL (2.124, 1.225–3.682, *p* = 0.007), and AFP ≥ 15 ng/mL (15.961, 7.890–32.285, *p* < 0.001) were independent risk factors. In contrast, total cholesterol > 240 mg/dL was associate d with a decreased risk (0.278, 0.086–0.898, *p* = 0.032). For HCV HCC development, old age (1.061, 1.033–1.089, *p* < 0.001), male gender (1.756, 1.195–2.581, *p* = 0.004), AST > 45 U/L (3.469, 2.222–5.499, *p* < 0.001), high FIB4 score (1.233, 1.198–1.269, *p* < 0.001), high blood sugar (1. 005, 1.000–1.09, *p* = 0.036), high platelet count < 150 × 10^3^ cells/µL (2.282, 1.471–3.540, *p* < 0.001), and AFP ≥ 15 ng/mL (2.956, 1.746–5.001, *p* < 0.001) were independent risk factors. For BC HCC development, male gender (HR: 5.813, 95% CI: 1.260–26.810, *p* = 0.024) and AFP ≥ 15 ng/mL (7.941, 2.119–29.756, *p* = 0.002) were independent risk factors.

### 3.3. Comparison of Clinical Features of HCC Patients among HBV, HCV, and NBNC Groups

Table 3 demonstrates the differences of the clinical features among the 35 patients with NBNC HCC, the 72 patients with HBV HCC, and the 109 patients with HCV HCC. Due to low case numbers and for the sake of simplicity, the BC HCC group was not included. Among the three groups, the NBNC HCC group had the highest platelet count, the HBV HCC group was the youngest, and the HCV HCC group had the highest AST, ALT levels, FIB4, and APRI scores. There were no significant differences among the three groups regarding sex, BMI, diabetes, hypertension, renal function, blood sugar, uric acid, total cholesterol, triglycerides, or AFP levels.

### 3.4. Incidence of HCC Stratified by Viral Etiology

**Figure 2:**
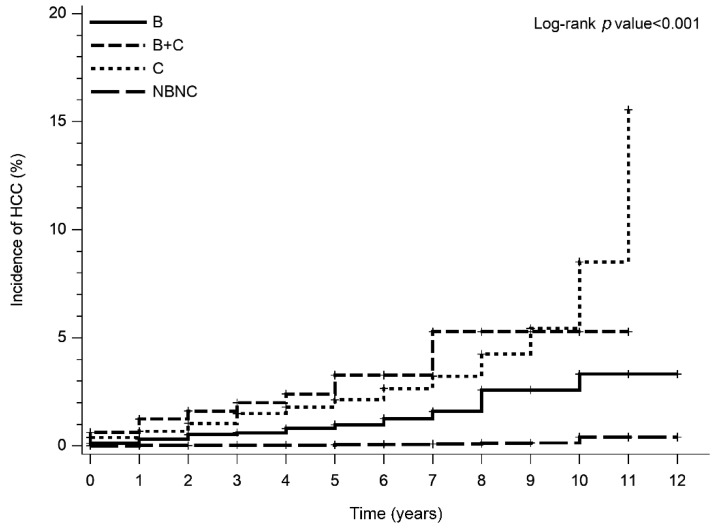
Cumulative incidence of HCC stratified by etiology.

A total of 228 participants developed HCC during the follow-up period. Figure 2 demonstrates the cumulative incidence of HCC among the different etiologies. The NBNC group had the lowest cumulative incidence of HCC compared to the other groups (*p* < 0.001).

### 3.5. All-Cause Mortality Stratified by Viral Etiology

**Figure 3:**
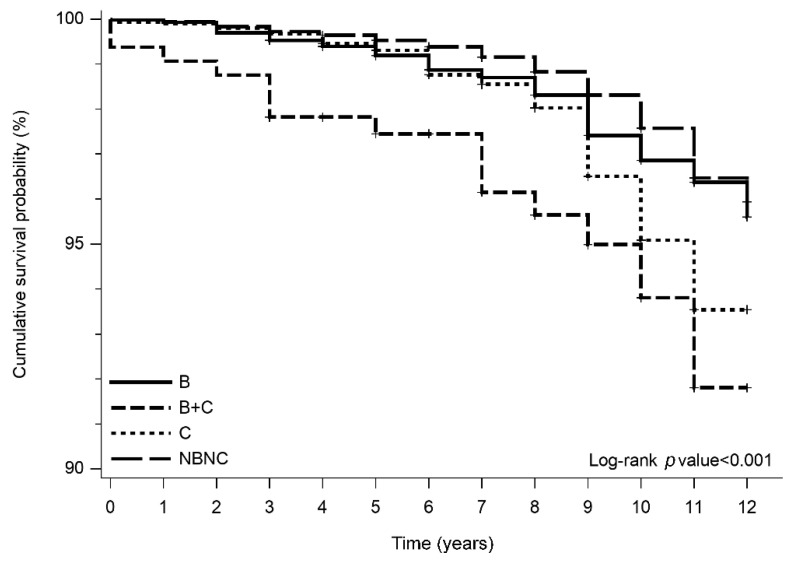
Cumulative survival probability stratified by etiology.

A total of 926 deaths were encountered during the follow-up period, with 98 of the deaths attributable to obvious hepatic events. Among the total deaths, 101 were in the HBV group (1.9%), 109 were in the HCV group (3.5%), 15 were in the BC group (4.7%), and 701 were in the NBNC group (1.6%). As shown in Figure 3, Kaplan–Meier analysis revealed that the NBNC group had a higher cumulative probability of survival compared to other groups (*p* < 0.001).

## 4. Discussion

In addition to chronic viral hepatitis infections, several metabolic, environmental, and genetic factors are associated with HCC. It is important to identify the risk factors of HCC for specific populations, as the efficaciousness and cost-effectiveness of chemoprevention efforts in HCC depend on surveillance programs with algorithms to identify and enroll priority at-risk patients. One good example is the use of an algorithm incorporating biomarkers such as AFP, AST, ALT, and platelet counts and clinical features such as age and DM to achieve a risk-score-guided invitation, which resulted in a reduction of HCC mortality by more than 30% [25]. Although the population attributable fractions of these non-viral factors included in the algorithm have been well scrutinized by several clinical observations [3,8], high-quality, population-based studies investigating the association between risk factors and NBNC HCC are sparse.

In contrast to most previous studies that were retrospective and hospital-based, our study inspected the risk factors of HCC in a prospective manner from a large community cohort by connecting patient data to a national database. After a median follow-up of 6 years, we observed that male gender and AFP ≥ 15 ng/mL were universal risk factors for HCC development, irrespective of underlying hepatitis virus infection status. Other common risk factors for HCC development included old age, high AST, low platelet count, and high FIB4 score. Notably, high BMI, high blood sugar, and low TG were independent risk factors for HCC development exclusively in the NBNC group. Although DM has been associated with HCC development in most studies [8,9,10,11,13,14,18], in the present study this relationship was not significant for all groups of participants. Instead, fasting blood sugar was associated with HCC development in the NBNC group. Underestimation of DM may be a possible explanation, as this diagnosis was obtained by participant interviews.

In this study, the HBV and HCV groups had similar characteristics for those with HCC development, including old age; male gender; higher serum creatinine, total cholesterol, AST, ALT, AFP levels, and APRI and FIB4 scores; and lower platelet counts compared to their non-HCC counterparts (Supplementary Tables S1 and S2). When compared to NBNC participants for whom high BMI, high sugar, and low TG levels were risk factors, these three factors were not linked to HCC development in the HBV or HCV group. Since advanced liver fibrosis is a well-established risk factor for HCC, two laboratory surrogates for degree of liver fibrosis were evaluated. Our results suggest that FIB4 score is superior to APRI for predicting HCC in community settings since the difference of FIB4 score was significant for all groups except for the BC HCC group, in comparison to no difference in APRI for all groups as determined by Cox proportional hazards analysis. Although high creatinine level was associated with HCC for both HBV and HCV groups, it was not associated with HCC in the NBNC group. This was in concordance with previous assumptions that HBV or HCV infection is possibly associated with increased risk of developing reduced glomerular filtration rate in the general population [26,27]. Notably, AFP < 15 ng/mL had a high negative predictive value, as AFP levels were <15 ng/mL in >97% of HCC-free participants, irrespective of their underlying viral hepatitis infection status (Table 1, Supplementary Tables S1–S3). This indicates that including AFP in health screening is cost-effective, especially in areas with low HCC prevalence, as close surveillance for liver cancer is mandated only for those with elevated AFP levels.

Because metabolic syndrome is a well-established risk factor for many malignancies (including HCC) and triglyceride > 150 mg/dL is among the diagnostic criteria for metabolic syndrome [28], it is reasonable to expect a positive association between the serum triglyceride level and HCC. Indeed, several retrospective, hospital-based studies have revealed this positive relationship between high triglyceride levels with NBNC HCC [11]. However, our results indicate that triglyceride levels > 150 mg/dL were associated with a decreased risk of HCC (HR: 0.216, 95% CI: 0.074–0.625, *p* = 0.005), which is consistent with another prospective study that also demonstrated an inverse relationship between hypertriglyceridemia with risk of NBNC HCC [14]. Although the exact mechanisms between low serum TG level and HCC risk deserves further investigation, the induction of lipogenic enzymes in HCCs may explain these contradictory results regarding serum triglyceride levels and HCC risk between prospective community-based and retrospective hospital-based studies [29]. These contradictory results also illustrate the importance of prospective studies in eliminating the potential biases elicited in retrospective studies.

Our study revealed that the presence of chronic viral hepatitis infections still poses the highest risk of HCC development compared to other nonviral risk factors. Besides, although the detailed causes of individual mortality were not available in this study, the higher accumulative mortality in HBV and HCV groups provided indirect support for the previous observation that showed increased mortality from hepatic and extrahepatic diseases with chronic HCV infection [27].

One major limitation of this study is the lack of information regarding past or occult HBV infection, as some studies indicated that NBNC HCCs are connected to past HBV infection in HBV-endemic or non-endemic areas [12,22,23] and other studies indicated that occult HBV infection increased the risk of NBNC HCC [30]. However, another study also demonstrated that the association between occult HBV infection and NBNC HCC was weak [24]. As the present study took advantage of a government-funded health-promotion program containing established investigation items which did not include anti-HBc and HBV DNA, it was not possible to obtain information regarding the proportion of patients with past or occult HBV infection. However, as the previous study showed that the prevalence of anti-HBc was approximately 80% in adults born prior to 1984 when the national HBV vaccination program was introduced in Taiwan [31], it is therefore speculated that approximately 80% of the participants in this study might be positive for anti-HBc. Another limitation for this study is that the status of alcohol use was not included. However, alcohol was reportedly associated with less than 1% of liver disease in Taiwan [32]. The other limitation is the lack of information on fatty liver disease. Nevertheless, risk factors for fatty liver disease in Taiwan including gender, age, BMI, diabetes, dyslipidemia, and hyperuricemia were included in this study [33]. Lastly, using anti-HCV to define NBNC HCC cannot distinguish past or present HCV infection, and the risk of developing HCC is significantly different between the two.

## 5. Conclusions

When compared to viral-hepatitis-related HCC, a better long-term outcome after curative surgery may be expected if NBNC HCC is diagnosed at an early stage [19]. Our study provided insights into the potential HCC risk factors which may shed some light on strategies for HCC prevention and surveillance for the general population in the era following hepatitis virus elimination. Further studies are needed to improve the surveillance programs with algorithms identifying and enrolling these priority at-risk patients.

## Figures and Tables

**Table 1:** Clinical characteristics of enrolled non-B, non-C participants.

	HCC	Non-HCC	
Variable	(n = 35)	(n = 43,510)	*p*-Value
Age (years)	65.0 ± 9.5	56.9 ± 10.0	<0.001
Sex			0.005
Male	23 (65.7%)	18,405 (42.3%)	
Female	12 (34.3%)	25,105 (57.7%)	
BMI (kg/m^2^)	25.8 ± 3.5	24.4 ± 3.5	0.019
<25	14 (40.0%)	26,269 (60.4%)	0.047
25–30	18 (51.4%)	14,560 (33.5%)	
≥30	3 (8.6%)	2681 (6.2%)	
DM			0.074
No	23 (65.7%)	34,019 (78.2%)	
Yes	12 (34.3%)	9491 (21.8%)	
HTN			0.386
No	24 (68.6%)	32,601 (74.9%)	
Yes	11 (31.4%)	10,909 (25.1%)	
GLU (mg/dL)	112.3 ± 35.7	96.8 ± 29.8	0.002
BUN (mg/dL)	16.5 ± 4.6	15.7 ± 4.5	0.284
CREA (mg/dL)	1.1 ± 0.2	1.1 ± 0.3	0.458
MDRD-eGFR	68.1 ± 11.9	68.4 ± 13.3	0.905
Renal function stage			0.766
1, 2	25 (71.4%)	32,042 (73.6%)	
3a, 3b, 4+	10 (28.6%)	11,468 (26.4%)	
UA (mg/dL)	6.3 ± 1.6	5.7 ± 1.6	0.028
TCHOL (mg/dL)	199.4 ± 47.6	211.2 ± 38.0	0.154
<240	26 (74.3%)	34,408 (79.1%)	0.486
≥240	9 (25.7%)	9102 (20.9%)	
TG (mg/dL)	103.8 ± 50.1	132.4 ± 99.0	0.002
<150	31 (88.6%)	31,354 (72.1%)	0.03
≥150	4 (11.4%)	12,156 (27.9%)	
HDL-C (mg/dL)	53.7 ± 13.6	57.7 ± 14.4	0.101
TCHOL/HDL	3.8 ± 0.8	3.8 ± 2.8	0.832
AFP (ng/mL)	1134.7 ± 6655.7	3.2 ± 1.6	0.322
<15	32 (91.4%)	43,475 (99.9%)	<0.001
≥15	3 (8.6%)	35 (0.1%)	
AST (U/L)	39.0 ± 30.7	24.9 ± 11.7	0.010
≤45	27 (77.1%)	42,097 (96.8%)	<0.001
>45	8 (22.9%)	1413 (3.2%)	
ALT (U/L)	34.2 ± 20.4	25.2 ± 18.4	0.004
≤45	29 (82.9%)	40,050 (92.0%)	0.056
>45	6 (17.1%)	3460 (8.0%)	
PLT (10^3^/µL)	202.6 ± 70.6	233.6 ± 60.7	0.003
<150	8 (22.9%)	2233 (5.1%)	<0.001
≥150	27 (77.1%)	41,277 (94.9%)	
APRI ^‡^	0.63 ± 0.85	0.29 ± 0.19	0.022
FIB4 ^§^	2.57 ± 2.23	1.38 ± 0.70	0.003

AFP: alpha fetoprotein; ALT: alanine aminotransferase; AST: aspartate aminotransferase; BMI: body mass index; BUN: blood urea nitrogen; CREA: creatinine; DM: diabetes mellitus; GLU: glucose; HDL-C: high-density lipoprotein cholesterol; HTN: hypertension; MDRD-eGFR: modification of diet in renal disease equation-estimated glomerular filtration rate; PLT: platelet; TCHOL: total cholesterol; TG: triglycerides; UA: uric acid. ^‡^ APRI = [(AST × 100)/(PLT × 40)]; ^§^ FIB4 = [(Age × AST)/(PLT × (ALT)^1/2^)].

**Table 2:** Univariate and multivariate Cox proportional hazards model for predictors of HCC in non-B, non-C participants.

	Univariable		Multivariable ^§^	
Variable	HR (95% CI)	*p*-Value	HR (95% CI)	*p*-Value
Age	1.081 (1.043, 1.120)	<0.001	1.058 (1.019, 1.098)	0.003
Sex				
Male vs. Female	2.787 (1.385, 5.608)	0.004	2.446 (1.200, 4.985)	0.014
BMI				
25–30 vs. <25	2.323 (1.155, 4.671)	0.018	2.480 (1.203, 5.112)	0.014
≥30 vs. <25	2.167 (0.622, 7.543)	0.224	2.519 (0.695, 9.132)	0.160
AST				
>45 vs. ≤45	9.330 (4.236, 20.550)	<0.001	6.816 (2.945, 15.779)	<0.001
ALT				
>45 vs. ≤45	2.570 (1.066, 6.191)	0.035		
BUN	1.021 (0.952, 1.095)	0.555		
CREA	1.302 (0.626, 2.705)	0.480		
Renal function stage				
3a, 3b, 4+ vs. 1, 2	0.958 (0.458, 2.006)	0.910		
UA	1.250 (1.035, 1.510)	0.021		
TCHOL				
≥240 vs. <240	1.291 (0.605, 2.757)	0.509		
TG				
≥150 vs. <150	0.351 (0.124, 0.994)	0.049	0.216 (0.074, 0.625)	0.005
HDL-C	0.981 (0.956, 1.007)	0.147		
TCHOL/HDL	0.943 (0.678, 1.311)	0.727		
AFP				
≥15 vs. <15	99.519 (30.377, 326.041)	<0.001	143.938 (43.094, 480.760)	<0.001
PLT				
<150 vs. ≥150	5.383 (2.440, 11.876)	<0.001		
DM				
Yes vs. No	1.678 (0.835, 3.375)	0.146		
HTN				
Yes vs. No	1.090 (0.533, 2.227)	0.814		
APRI ^‡^	1.524 (1.309, 1.775)	<0.001		
FIB4 ^†^	1.268 (1.196, 1.345)	<0.001	1.262 (1.154, 1.381)	<0.001
GLU	1.009 (1.003, 1.014)	0.001	1.009 (1.002, 1.015)	0.006

AFP: alpha fetoprotein; ALT: alanine aminotransferase; AST: aspartate aminotransferase; BMI: body mass index; BUN: blood urea nitrogen; CREA: creatinine; DM: diabetes mellitus; GLU: glucose; HDL-C: high-density lipoprotein cholesterol; HTN: hypertension; MDRD-eGFR: modification of diet in renal disease equation-estimated glomerular filtration rate; PLT: platelet; TCHOL: total cholesterol; TG: triglycerides; UA: uric acid. ^‡^ APRI = [(AST × 100)/(PLT × 40)]; ^†^ FIB4 = [(Age × AST)/(PLT × (ALT)^1/2^)]. ^§^ Associated factors which showed *p*-values < 0.5 in univariable analysis were entered into stepwise multivariable analysis.

**Table 3:** Comparison of the clinical features of HCC patients among HBV, HCV, and NBNC groups.

Variable	HBV (n = 72)	HCV (n = 109)	NBNC (n = 35)	*p*-Value	Significant Pairs from Bonferroni’s Pairwise Comparison ^§^
Age (years)	59.8 ± 9.9	67.1 ± 5.9	65.0 ± 9.5	<0.001	b > a, c > a
Sex				0.052	
Male	53 (73.6%)	61 (56.0%)	23 (65.7%)		
Female	19 (26.4%)	48 (44.0%)	12 (34.3%)		
BMI (kg/m^2^)	24.6 ± 3.4	24.4 ± 4.0	25.8 ± 3.5	0.146	
<25	41 (56.9%)	69 (63.3%)	14 (40.0%)	0.121	
25–30	26 (36.1%)	30 (27.5%)	18 (51.4%)		
≥30	5 (6.9%)	10 (9.2%)	3 (8.6%)		
DM				0.236	
No	58 (80.6%)	80 (73.4%)	23 (65.7%)		
Yes	14 (19.4%)	29 (26.6%)	12 (34.3%)		
HTN				0.092	
No	56 (77.8%)	68 (62.4%)	24 (68.6%)		
Yes	16 (22.2%)	41 (37.6%)	11 (31.4%)		
GLU (mg/dL)	100.9 ± 27.5	104.2 ± 40.2	112.3 ± 35.7	0.301	
BUN (mg/dL)	16.0 ± 4.3	17.7 ± 6.6	16.5 ± 4.6	0.120	
CREA (mg/dL)	1.1 ± 0.2	1.2 ± 0.3	1.1 ± 0.2	0.433	
MDRD-eGFR	68.7 ± 14.5	63.1 ± 14.6	68.1 ± 11.9	0.020	a > b
Renal function stage				0.096	
1, 2	51 (70.8%)	62 (56.9%)	25 (71.4%)		
3a, 3b, 4+	21 (29.2%)	47 (43.1%)	10 (28.6%)		
UA (mg/dL)	5.8 ± 1.2	6.3 ± 1.8	6.3 ± 1.6	0.100	
TCHOL (mg/dL)	186.5 ± 37.3	175.3 ± 36.7	199.4 ± 47.6	0.004	c > b
<240	69 (95.8%)	104 (95.4%)	26 (74.3%)	<0.001	
≥240	3 (4.2%)	5 (4.6%)	9 (25.7%)		
TG (mg/dL)	104.2 ± 61.9	105.7 ± 61.0	103.8 ± 50.1	0.981	
<150	61 (84.7%)	92 (84.4%)	31 (88.6%)	0.826	
≥150	11 (15.3%)	17 (15.6%)	4 (11.4%)		
HDL-C (mg/dL)	61.0 ± 19.2	52.5 ± 15.4	53.7 ± 13.6	0.003	a > b
TCHOL/HDL	3.3 ± 1.0	3.5 ± 1.1	3.8 ± 0.8	0.034	c > a
AFP (ng/mL)	214.0 ± 1230.4	18.4 ± 65.0	1134.7 ± 6655.7	0.114	
<15	62 (86.1%)	89 (81.7%)	32 (91.4%)	0.347	
≥15	10 (13.9%)	20 (18.3%)	3 (8.6%)		
AST (U/L)	50.4 ± 53.2	78.8 ± 52.1	39.0 ± 30.7	<0.001	b > a, b > c
≤45	54 (75.0%)	34 (31.2%)	27 (77.1%)	<0.001	
>45	18 (25.0%)	75 (68.8%)	8 (22.9%)		
ALT (U/L)	47.2 ± 46.9	87.9 ± 61.0	34.2 ± 20.4	<0.001	b > a, b > c
≤45	51 (70.8%)	30 (27.5%)	29 (82.9%)	<0.001	
>45	21 (29.2%)	79 (72.5%)	6 (17.1%)		
PLT (10^9^/L)	160.2 ± 56.5	144.8 ± 56.4	202.6 ± 70.6	<0.001	c > a, c > b
<150	30 (41.7%)	66 (60.6%)	8 (22.9%)	<0.001	
≥150	42 (58.3%)	43 (39.4%)	27 (77.1%)		
APRI ^‡^	0.97 ± 1.18	1.67 ± 1.42	0.63 ± 0.85	<0.001	b > a, b > c
FIB4 ^†^	3.18 ± 2.45	4.67 ± 2.99	2.57 ± 2.23	<0.001	b > a, b > c

BMI: body mass index; DM: diabetes mellitus; HTN: hypertension; GLU: glucose; BUN: blood urea nitrogen; CREA: creatinine; UA: uric acid; TCHOL: total cholesterol; TG: triglycerides; HDL-C: high-density lipoprotein cholesterol; AFP: alpha fetoprotein; AST: aspartate aminotransferase; ALT: alanine aminotransferase; PLT: platelet. ^‡^ APRI = [(AST × 100)/(PLT × 40)]; ^†^ FIB4 = [(Age × AST)/(PLT × (ALT)^1/2^)]. ^§^ a = HBV, b = HCV, c = NBNC.

## Data Availability

The data presented in this study are available on reasonable request to the corresponding author.

## Supplementary Materials

The following supporting information can be downloaded at: https://www.mdpi.com/article/10.3390/v14050984/s1, Table S1. Clinical characteristics of enrolled HBV participants; Table S2. Clinical characteristics of enrolled HCV participants; Table S3. Clinical characteristics of enrolled dual HBV and HCV participants; Table S4. Univariate and multivariate Cox proportional hazards model for predictors of HCC in HBV participants; Table S5. Univariate and multivariate Cox proportional hazards model for predictors of HCC in HCV participants; Table S6. Univariate and multivariate Cox proportional hazards model for predictors of HCC in dual HBV and HCV participants.
