# Supplementary material for: Non-B, Non-C Hepatocellular Carcinoma in an HBV- and HCV-Endemic Area: A Community-Based Prospective Longitudinal Study
Source: Viruses. 2022 May 7;14(5):984. doi: 10.3390/v14050984 (PMC9145519; doi:10.3390/v14050984)
Supplement: Supplementary file 1 [file viruses-14-00984-s001.zip › viruses-1665856-supplementary.pdf]

Supplementary Table S1. Clinical characteristics of enrolled HBV participants.

Variable	HCC (n=72)	Non-HCC (n=5601)	*p*-Value
Age (years)	59.8±9.9	55.0±9.5	<0.001
Sex			<0.001
Male	53 (73.6%)	2609 (46.6%)	
Female	19 (26.4%)	2992 (53.4%)	
BMI (kg/m^2)	24.6±3.4	24.4±3.6	0.635
<25	41 (56.9%)	3405 (60.8%)	0.802
25-30	26 (36.1%)	1843 (32.9%)	
≥30	5 (6.9%)	353 (6.3%)	
DM			0.943
No	58 (80.6%)	4493 (80.2%)	
Yes	14 (19.4%)	1108 (19.8%)	
HTN			0.950
No	56 (77.8%)	4339 (77.5%)	
Yes	16 (22.2%)	1262 (22.5%)	
GLU (mg/dL)	100.9±27.5	96.0±30.1	0.170
BUN (mg/dL)	16.0±4.3	15.2±4.3	0.128
CREA (mg/dL)	1.1±0.2	1.0±0.2	0.005
MDRD-eGFR	68.7±14.5	70.0±12.9	0.412
Renal function stage			0.108
1, 2	51 (70.8%)	4406 (78.7%)	
3a, 3b, 4+	21 (29.2%)	1195 (21.3%)	
UA (mg/dL)	5.8±1.2	5.7±1.5	0.269
TCHOL (mg/dL)	186.5±37.3	202.1±35.9	<0.001
<240	69 (95.8%)	4820 (86.1%)	0.017
≥240	3 (4.2%)	781 (13.9%)	
TG (mg/dL)	104.2±61.9	115.2±82.4	0.141
<150	61 (84.7%)	4467 (79.8%)	0.297
≥150	11 (15.3%)	1134 (20.2%)	
HDL-C (mg/dL)	61.0±19.2	58.3±14.8	0.250
TCHOL/HDL	3.3±1.0	3.6±1.0	0.002
AFP (ng/mL)	214.0±1230.4	3.2±2.4	0.150
<15	62 (86.1%)	5581 (99.6%)	<0.001
≥15	10 (13.9%)	20 (0.4%)	
AST (U/L)	50.4±53.2	28.6±19.9	<0.001
≤45	54 (75.0%)	5227 (93.3%)	<0.001
>45	18 (25.0%)	374 (6.7%)	
ALT (U/L)	47.2±46.9	31.0±35.2	0.005
≤45	51 (70.8%)	4904 (87.6%)	<0.001
>45	21 (29.2%)	697 (12.4%)	
PLT (10^3/μL)	160.2±56.5	214.2±57.3	<0.001
<150	30 (41.7%)	613 (10.9%)	<0.001
≥150	42 (58.3%)	4988 (89.1%)	
APRI[†]	0.97±1.18	0.37±0.34	<0.001
FIB4 [‡]	3.18±2.45	1.53±0.87	<0.001

AFP: alpha fetoprotein; ALT: alanine aminotransferase; AST: aspartate aminotransferase; BMI: body mass index; BUN: blood urea nitrogen; CREA: creatinine; DM: diabetes mellitus; GLU: glucose; HDL-C: high-density lipoprotein cholesterol; HTN: hypertension; MDRD-eGFR: modification of diet in renal disease equation-estimated glomerular filtration rate; PLT: platelet; TCHOL: total cholesterol; TG: triglycerides; UA: uric acid.

†APRI = [(AST×100) / (PLT×40)]; ‡FIB4 = [(Age×AST) / (PLT×$(ALT)^{1/2}$)].

Supplementary Table S2. Clinical characteristics of enrolled HCV participants.

Variable	HCC (n=109)	Non-HCC (n=2993)	*p*-Value
Age (years)	67.1±5.9	61.8±9.2	<0.001
Sex			<0.001
Male	61 (56.0%)	1134 (37.9%)	
Female	48 (44.0%)	1859 (62.1%)	
BMI (kg/m^2)	24.4±4.0	24.5±3.6	0.825
<25	69 (63.3%)	1778 (59.4%)	0.221
25-30	30 (27.5%)	1026 (34.3%)	
≥30	10 (9.2%)	189 (6.3%)	
DM			0.462
No	80 (73.4%)	2288 (76.4%)	
Yes	29 (26.6%)	705 (23.6%)	
HTN			0.024
No	68 (62.4%)	2164 (72.3%)	
Yes	41 (37.6%)	829 (27.7%)	
GLU (mg/dL)	104.2±40.2	99.8±33.1	0.257
BUN (mg/dL)	17.7±6.6	16.5±5.0	0.046
CREA (mg/dL)	1.2±0.3	1.1±0.3	0.002
MDRD-eGFR	63.1±14.6	66.0±13.7	0.031
Renal function stage			0.061
1, 2	62 (56.9%)	1963 (65.6%)	
3a, 3b, 4+	47 (43.1%)	1030 (34.4%)	
UA (mg/dL)	6.3±1.8	5.8±1.5	0.004
TCHOL (mg/dL)	175.3±36.7	193.2±37.9	<0.001
<240	104 (95.4%)	2651 (88.6%)	0.026
≥240	5 (4.6%)	342 (11.4%)	
TG (mg/dL)	105.7±61.0	109.4±70.4	0.588
<150	92 (84.4%)	2495 (83.4%)	0.774
≥150	17 (15.6%)	498 (16.6%)	
HDL-C (mg/dL)	52.5±15.4	55.6±14.2	0.026
TCHOL/HDL	3.5±1.1	3.6±0.9	0.377
AFP (ng/mL)	18.4±65.0	3.9±4.2	0.022
<15	89 (81.7%)	2947 (98.5%)	<0.001
≥15	20 (18.3%)	46 (1.5%)	

AST (U/L)	78.8±52.1	41.1±48.4	<0.001
≤45	34 (31.2%)	2297 (76.7%)	<0.001
>45	75 (68.8%)	696 (23.3%)	
ALT (U/L)	87.9±61.0	46.2±62.9	<0.001
≤45	30 (27.5%)	2116 (70.7%)	<0.001
>45	79 (72.5%)	877 (29.3%)	
PLT (10^3/μL)	144.8±56.4	195.6±58.9	<0.001
<150	66 (60.6%)	636 (21.2%)	<0.001
≥150	43 (39.4%)	2357 (78.8%)	
APRI†	1.67±1.42	0.63±0.90	<0.001
FIB4 ‡	4.67±2.99	2.24±1.54	<0.001

AFP: alpha fetoprotein; ALT: alanine aminotransferase; AST: aspartate aminotransferase; BMI: body mass index; BUN: blood urea nitrogen; CREA: creatinine; DM: diabetes mellitus; GLU: glucose; HDL-C: high-density lipoprotein cholesterol; HTN: hypertension; MDRD-eGFR: modification of diet in renal disease equation-estimated glomerular filtration rate; PLT: platelet; TCHOL: total cholesterol; TG: triglycerides; UA: uric acid.

†APRI = [(AST×100) / (PLT×40)]; ‡FIB4 = [(Age×AST) / (PLT×$(ALT)^{1/2}$)].

Supplementary Table S3. Clinical characteristics of enrolled dual HBV and HCV participants.

Variable	HCC (n=12)	Non-HCC (n=310)	*p*-Value
Age (years)	60.5±6.0	59.6±9.3	0.728
Sex			0.005
Male	12 (100.0%)	132 (42.6%)	
Female		178 (57.4%)	
BMI (kg/m^2)	23.4±3.7	24.9±3.9	0.200
<25	8 (66.7%)	163 (52.6%)	0.738
25-30	4 (33.3%)	120 (38.7%)	
≥30		27 (8.7%)	
DM			0.310
No	12 (100.0%)	238 (76.8%)	
Yes		72 (23.2%)	
HTN			0.523
No	12 (100.0%)	224 (72.3%)	
Yes		86 (27.7%)	
GLU (mg/dL)	106.3±37.7	99.2±33.3	0.472
BUN (mg/dL)	15.9±3.6	16.5±5.5	0.682
CREA (mg/dL)	1.1±0.3	1.1±0.3	0.395
MDRD-eGFR	71.4±26.7	67.7±15.1	0.639
Renal function stage			0.196
1, 2	7 (58.3%)	232 (74.8%)	
3a, 3b, 4+	5 (41.7%)	78 (25.2%)	

UA (mg/dL)	6.7±2.3	5.8±1.6	0.222
TCHOL (mg/dL)	204.0±50.8	196.5±37.0	0.500
<240	9 (75.0%)	269 (86.8%)	0.217
≥240	3 (25.0%)	41 (13.2%)	
TG (mg/dL)	160.3±161.8	111.1±69.9	0.317
<150	8 (66.7%)	254 (81.9%)	0.247
≥150	4 (33.3%)	56 (18.1%)	
HDL-C (mg/dL)	53.6±16.6	57.3±15.1	0.398
TCHOL/HDL	4.0±1.3	3.6±1.0	0.126
AFP (ng/mL)	37.6±71.7	4.5±13.5	0.139
<15	9 (75.0%)	301 (97.1%)	0.007
≥15	3 (25.0%)	9 (2.9%)	
AST (U/L)	61.8±46.0	40.8±30.1	0.146
≤45	6 (50.0%)	244 (78.7%)	0.03
>45	6 (50.0%)	66 (21.3%)	
ALT (U/L)	73.8±51.2	47.4±46.8	0.057
≤45	4 (33.3%)	220 (71.0%)	0.009
>45	8 (66.7%)	90 (29.0%)	
PLT $10^3/\mu L$)	174.9±65.8	192.8±56.6	0.287
<150	4 (33.3%)	70 (22.6%)	0.482
≥150	8 (66.7%)	240 (77.4%)	
APRI[†]	0.95±0.64	0.63±0.67	0.107
FIB4 [‡]	2.74±1.53	2.11±1.28	0.099

AFP: alpha fetoprotein; ALT: alanine aminotransferase; AST: aspartate aminotransferase; BMI: body mass index; BUN: blood urea nitrogen; CREA: creatinine; DM: diabetes mellitus; GLU: glucose; HDL-C: high-density lipoprotein cholesterol; HTN: hypertension; MDRD-eGFR: modification of diet in renal disease equation-estimated glomerular filtration rate; PLT: platelet; TCHOL: total cholesterol; TG: triglycerides; UA: uric acid.

[†]APRI = [(AST×100) / (PLT×40)]; [‡]FIB4 = [(Age×AST) / (PLT×$(ALT)^{1/2}$)].

Supplementary Table S4. Univariate and multivariate Cox proportional hazards model for predictors of HCC in HBV participants.

	Univariable		Multivariable[§]	
Variable	HR (95% CI)	p-value	HR (95% CI)	*p*-Value
Age	1.047 (1.022, 1.073)	<0.001		
Sex				
Male vs. Female	3.200 (1.894, 5.406)	<0.001	2.848 (1.628, 4.982)	<0.001
BMI				
25-30 vs. <25	1.190 (0.728, 1.945)	0.488		
≥30 vs. <25	1.232 (0.487, 3.117)	0.660		
AST				
>45 vs. ≤45	5.171 (3.029, 8.828)	<0.001		
ALT				
>45 vs. ≤45	3.073 (1.847, 5.113)	<0.001		
BUN	1.016 (0.965, 1.069)	0.543		
CREA	2.310 (1.150, 4.642)	0.019		
Renal function stage				
3a, 3b, 4+ vs. 1, 2	1.196 (0.718, 1.993)	0.492		
UA	1.056 (0.912, 1.223)	0.466		
TCHOL				
≥240 vs. <240	0.266 (0.084, 0.846)	0.025	0.278 (0.086, 0.898)	0.032
TG				
≥150 vs. <150	0.746 (0.393, 1.418)	0.372		
HDL-C	1.011 (0.997, 1.026)	0.128	1.024 (1.009, 1.040)	0.002
TCHOL/HDL	0.627 (0.471, 0.836)	0.001		
AFP				
≥15 vs. <15	39.493 (20.175, 77.308)	<0.001	15.961 (7.890, 32.285)	<0.001
PLT				
≥150 vs. <150	5.358 (3.352, 8.564)	<0.001	2.124 (1.225, 3.682)	0.007
DM				
Yes vs. No	0.888 (0.495, 1.592)	0.690		
HTN				
Yes vs. No	0.780 (0.447, 1.361)	0.382		
APRI[†]	1.794 (1.613, 1.995)	<0.001		
FIB4 [‡]	1.347 (1.286, 1.411)	<0.001	1.283 (1.197, 1.375)	<0.001
GLU	1.004 (0.999, 1.008)	0.128		

AFP: alpha fetoprotein; ALT: alanine aminotransferase; AST: aspartate aminotransferase; BMI: body mass index; BUN: blood urea nitrogen; CREA: creatinine; DM: diabetes mellitus; GLU: glucose; HDL-C: high-density lipoprotein cholesterol; HTN: hypertension; MDRD-eGFR: modification of diet in renal disease equation-estimated glomerular filtration rate; PLT: platelet; TCHOL: total cholesterol; TG: triglycerides; UA: uric acid.

[†]APRI = [(AST×100) / (PLT×40)]; [‡]FIB4 = [(Age×AST) / (PLT×$(ALT)^{1/2}$)].

[§]: Associated factors which showed *p*-values < 0.5 in univariable analysis were entered into stepwise multivariable analysis.

Supplementary Table S5. Univariate and multivariate Cox proportional hazards model for predictors of HCC in HCV participants.

	Univariable		Multivariable§	
Variable	HR (95% CI)	*p*-Value	HR (95% CI)	*p*-Value
Age	1.076 (1.050, 1.103)	<0.001	1.061 (1.033, 1.089)	<0.001
Sex				
Male vs. Female	2.030 (1.390, 2.965)	<0.001	1.756 (1.195, 2.581)	0.004
BMI				
25-30 vs. <25	0.751 (0.489, 1.153)	0.190		
≥30 vs. <25	1.582 (0.814, 3.073)	0.176		
AST				
>45 vs. ≤45	6.795 (4.531, 10.190)	<0.001	3.496 (2.222, 5.499)	<0.001
ALT				
>45 vs. ≤45	5.897 (3.872, 8.980)	<0.001		
BUN	1.039 (1.006, 1.072)	0.020		
CREA	1.680 (1.244, 2.269)	<0.001		
Renal function stage				
3a, 3b, 4+ vs. 1, 2	1.276 (0.871, 1.868)	0.211		
UA	1.254 (1.121, 1.403)	<0.001		
TCHOL				
≥240 vs. <240	0.350 (0.143, 0.858)	0.022		
TG				
≥150 vs. <150	0.996 (0.593, 1.671)	0.987		
HDL-C	0.985 (0.971, 0.999)	0.042		
TCHOL/HDL	0.869 (0.702, 1.076)	0.198		
AFP				
≥15 vs. <15	12.025 (7.397, 19.549)	<0.001	2.956 (1.746, 5.007)	<0.001
PLT				
≥150 vs. <150	5.561 (3.782, 8.176)	<0.001	2.282 (1.471, 3.540)	<0.001
DM				
Yes vs. No	1.121 (0.733, 1.715)	0.598		
HTN				
Yes vs. No	1.384 (0.939, 2.040)	0.101		
APRI†	1.145 (1.106, 1.186)	<0.001		
FIB4 ‡	1.233 (1.198, 1.269)	<0.001	1.101 (1.041, 1.164)	<0.001
GLU	1.005 (1.000, 1.009)	0.031	1.005 (1.000, 1.009)	0.036

AFP: alpha fetoprotein; ALT: alanine aminotransferase; AST: aspartate aminotransferase; BMI: body mass index; BUN: blood urea nitrogen; CREA: creatinine; DM: diabetes mellitus; GLU: glucose; HDL-C: high-density lipoprotein cholesterol; HTN: hypertension; MDRD-eGFR: modification of diet in renal disease equation-estimated glomerular filtration rate; PLT: platelet; TCHOL: total cholesterol; TG: triglycerides; UA: uric acid.

†APRI = [(AST×100) / (PLT×40)]; ‡FIB4 = [(Age×AST) / (PLT×$(ALT)^{1/2}$)].

§: Associated factors which showed *p*-values < 0.5 in univariable analysis were entered into stepwise multivariable analysis.

Supplementary Table S6. Univariate and multivariate Cox proportional hazards model for predictors of HCC in dual HBV and HCV participants.

	Univariable		Multivariable§	
Variable	HR (95% CI)	*p*-Value	HR (95% CI)	*p*-Value
Age	1.014 (0.951, 1.082)	0.670		
Sex				
Male vs. Female	6.706 (1.469, 30.611)	0.014	5.813 (1.260, 26.810)	0.024
BMI				
25-30 vs. <25	0.710 (0.214, 2.358)	0.576		
≥30 vs. <25	0.000 (0.000, -)	0.994		
AST				
>45 vs. ≤45	3.847 (1.240, 11.939)	0.020		
ALT				
>45 vs. ≤45	5.114 (1.538, 17.002)	0.008		
BUN	0.967 (0.856, 1.093)	0.590		
CREA	1.612 (0.497, 5.226)	0.426		
Renal function stage				
3a, 3b, 4+ vs. 1, 2	1.804 (0.572, 5.695)	0.314		
UA	1.301 (0.980, 1.729)	0.069		
TCHOL				
≥240 vs. <240	2.163 (0.585, 7.988)	0.247		
TG				
≥150 vs. <150	2.153 (0.648, 7.153)	0.211		
HDL-C	0.982 (0.941, 1.025)	0.402		
TCHOL/HDL	1.491 (0.909, 2.447)	0.114		
AFP				
≥15 vs. <15	10.578 (2.850, 39.267)	<0.001	7.941 (2.119, 29.756)	0.002
PLT				
≥150 vs. <150	1.568 (0.472, 5.208)	0.463		
DM				
Yes vs. No	0.291 (0.038, 2.258)	0.238		
HTN				
Yes vs. No	0.456 (0.100, 2.083)	0.311		
APRI†	1.497 (0.929, 2.412)	0.097		
FIB4 ‡	1.344 (0.988, 1.830)	0.060		
GLU	1.006 (0.994, 1.019)	0.304		

AFP: alpha fetoprotein; ALT: alanine aminotransferase; AST: aspartate aminotransferase; BMI: body mass index; BUN: blood urea nitrogen; CREA: creatinine; DM: diabetes mellitus; GLU: glucose; HDL-C: high-density lipoprotein cholesterol; HTN: hypertension; MDRD-eGFR: modification of diet in renal disease equation-estimated glomerular filtration rate; PLT: platelet; TCHOL: total cholesterol; TG: triglycerides; UA: uric acid.

†APRI = [(AST×100) / (PLT×40)]; ‡FIB4 = [(Age×AST) / (PLT×$(ALT)^{1/2}$)].

§: Associated factors which showed *p*-values < 0.5 in univariable analysis were entered into stepwise multivariable analysis.
